# Humoral response to mRNA‐based COVID‐19 vaccine in patients with de novo and pre‐existing immune thrombocytopenia with exacerbation of thrombocytopenia after vaccination

**DOI:** 10.1111/bjh.18447

**Published:** 2022-09-12

**Authors:** Akio Mori, Masahiro Onozawa, Mirei Kobayashi, Shihori Tsukamoto, Takashi Ishio, Emi Yokoyama, Koh Izumiyama, Makoto Saito, Haruna Muraki, Masanobu Morioka, Takanori Teshima, Takeshi Kondo

**Affiliations:** ^1^ Blood Disorders Center Aiiku Hospital Sapporo Japan; ^2^ Department of Hematology Hokkaido University Faculty of Medicine Sapporo Japan; ^3^ Division of Laboratory Aiiku Hospital Sapporo Japan; ^4^ Sapporo Clinical Laboratory, Inc. Sapporo Japan

Vaccination has been an effective public health measure in the coronavirus disease‐19 (COVID‐19) pandemic.^1^, ^2^ However, since the sudden death of a previously healthy recipient diagnosed with de novo immune thrombocytopenia (ITP) after vaccination was reported, public alarm has been heightened.^3^ Although it is difficult to precisely assess whether post‐vaccination thrombocytopenia is vaccine‐induced or incidental, further, the association is chronological and not necessarily causal, ITP can develop after COVID‐19 vaccination.^4^, ^5^, ^6^, ^7^ However, the humoral response in such patients is unknown and it must be of great concern to both patients and their physicians. Furthermore, a recent study reported that active treatment with corticosteroids reduced immunogenicity in patients with autoimmune cytopenias including ITP.^8^ Hence, we considered the possibility that corticosteroids treatment for patients with post‐COVID‐19 vaccine ITP exacerbation might affect the vaccine response. Therefore, in this prospective observation study, we investigated the antibody titres against severe acute respiratory syndrome coronavirus‐2 (SARS‐CoV‐2) in patients with de novo and pre‐existing ITP with exacerbation of thrombocytopenia after vaccination.

As a pre‐existing ITP cohort, 49 consecutive patients who had previously been diagnosed with ITP and had received two doses of a mRNA‐based COVID‐19 vaccine, either BNT162b2 or mRNA‐1273, were identified at Aiiku Hospital between 1 June 2021 and 31 March 2022. The diagnosis and the response criteria of ITP were defined according to International Consensus Guidelines.^9^ In patients with pre‐existing ITP, ‘exacerbation of ITP’ was defined as previously reported.^4^ Patients with pre‐existing ITP with exacerbation of thrombocytopenia within 30 days of the previous vaccination were considered as patients with post‐vaccine ITP exacerbation. Apart from this cohort, we had two additional patients with de novo ITP following COVID‐19 vaccination. Anti‐SARS‐CoV‐2 spike (S) antibodies were measured at 3 months ± 2 weeks, 6 months ± 4 weeks and 9 months ± 4 weeks after the second vaccine dose as previously described.^10^ Considering the mean age of the patients with ITP, we recruited healthcare workers aged ≥55 years who had received two doses of BNT162b2 vaccine as healthy controls (HCs) (*n* = 28). There was no significant difference in age between the patients with ITP and HCs (mean [SD] 66.6 [7.2] vs. 60.2 [4.4] years; *p* = 0.060). Individuals with a known history of COVID‐19 were excluded from both cohorts of patients and HCs.

Of the 49 consecutive patients with pre‐existing ITP, three patients (6.1%) showed exacerbation of ITP after COVID‐19 vaccination (Table 1). One patient (unique patient number [UPN] 1) had repeated thrombocytopenia after the second and third doses. Two additional patients with de novo ITP following COVID‐19 vaccination were identified in the same period (Table 1). Totally, four cases of exacerbation of ITP in three patients with pre‐existing ITP and two cases of de novo ITP were identified as cases of ITP after COVID‐19 vaccination. All except one of the patients required prompt treatment and all rescue treatments included corticosteroids. All of the treated patients responded and were on active treatment on the day of the data cut‐off or last observation.

**TABLE 1:** Patient demographics and outcomes

Onset type	UPN	Age, years	Sex	Treatment line(s) at the time of initial vaccination	Doses of vaccines at the onset of ITP	Onset of exacerbation of ITP, days	Treatment at the onset of vaccine‐associated ITP	Vaccine type of 1st and 2nd doses	Vaccine type of 3rd dose	Lowest platelet count after vaccination, ×10^9^/l	Bleeding symptoms	Initial and consecutive rescue treatments	Rescue treatments on the day of data cut‐off or last observation (days after treatments)	Days until platelets >30 × 10^9^/l after treatment	Response to rescue treatment	Antibody titres 3 months after vaccination, u/ml	Antibody titres 6 months after vaccination, u/ml	Antibody titres 9 months after vaccination, u/ml
Median		74	‐	2	2	15^a^	‐	‐	‐	9	‐	‐	‐	7	‐	‐	‐	‐
Pre‐existing	1	83	F	0	2	11	Untreated	BNT162b2	BNT162b2	7	Ecchymoses	HD‐DXM followed by PSL 30 mg, EPAG 12.5 mg	PSL 10 mg, EPAG 25 mg (233^b^)	6	CR	153	66.9	5020.0
					3	19	PSL 7.5 mg, EPAG 25 mg			19	No	PSL 30 mg, EPAG 50 mg	PSL 25 mg, Romiplostim 600 μg (43)	33	R			
	2	57	M	5	2	24	PSL 5 mg, EPAG 50 mg	mRNA‐1273	Not yet	11	Petechiae	PSL 30 mg, EPAG 50 mg	PSL 15 mg, EPAG 50 mg (191)	7	R	208	76.9	Not yet
	3	79	F	2	3	11	PSL 3 mg, EPAG 25 mg	BNT162b2	BNT162b2	5	Petechiae	HD‐DXM followed by PSL 30 mg, EPAG 37.5 mg	PSL 30 mg, EPAG 37.5 mg (14)	5	CR	250	74.7	18 602.0
De novo	4	74	M	‐	1	2	‐	BNT162b2	Not yet	3	Petechiae, ecchymoses, oral blood blisters	HD‐DXM followed by PSL 30 mg	PSL 6 mg (170)	11	CR	2.7	0.7	Not yet
	5	66	F	‐	2	20	‐	BNT162b2	BNT162b2	17	Ecchymoses	Untreated	Untreated	‐	‐	nd	1232.0	21554.0

Abbreviations: CR, complete response; EPAG, eltrombopag; F, female; HD‐DXM, high‐dose dexamethasone; ITP, immune thrombocytopenia; M, male; nd, no data; PSL, prednisolone; R, response; UPN, unique patient number.

^a^ There were two patients with post‐vaccine ITP exacerbation who were diagnosed ≥20 days after vaccination because they did not visit the hospital despite bleeding tendency.

^b^ Days after treatment to third vaccination.

Seroconversion rates after vaccination for both patients with post‐vaccine ITP exacerbation and HCs were 100%. However, the antibody titres in patients with post‐vaccine ITP exacerbation treated with corticosteroids were significantly lower than those in HCs at both 3 months (median [interquartile range, IQR] 153.0 [77.9–180.5] vs. 945.0 [402.5–1358.0] u/ml, *p* < 0.05) and 6 months (median [IQR] 66.9 [33.8–71.9] vs. 582.5 [240.5–1033.3] u/ml, *p* < 0.01) after vaccination (Figure 1A,B). On the other hand, in one patient with post‐vaccine ITP exacerbation who did not receive rescue treatment (UPN5), the antibody titre at 6 months after vaccination (1232.0 u/ml) was comparable to that in HCs (median: 582.5 u/ml). Furthermore, in a previously healthy individual who developed ITP immediately after the first dose (UPN4), the antibody titre at 3 months was positive but was relatively low (2.7 u/ml) and subsequently became seronegative. In this patient, high‐dose dexamethasone therapy followed by prednisolone was started immediately after the onset of ITP. It was recently reported that active therapy with corticosteroids reduced seroconversion rates and antibody titres after COVID‐19 vaccination in patients with autoimmune cytopenias including ITP and in patients with haematological disease in the setting of post‐allogenic stem cell transplantation.^8^, ^11^ In our study, although the number of cases is too small, treatment with corticosteroids may reduce the vaccine response in patients with post‐vaccine ITP exacerbation. Strict measures for prevention of infection such as social‐distancing practices might be recommended in patients with post‐vaccine ITP exacerbation who receive corticosteroids treatment. On the other hand, the seroconversion rate after a single‐dose of vaccine was shown to be lower than that after two doses of vaccine in immunocompromised patients.^12^, ^13^ Therefore, single‐dose vaccination may also have contributed to the lower antibody titres in patient UPN4.

**FIGURE 1:**
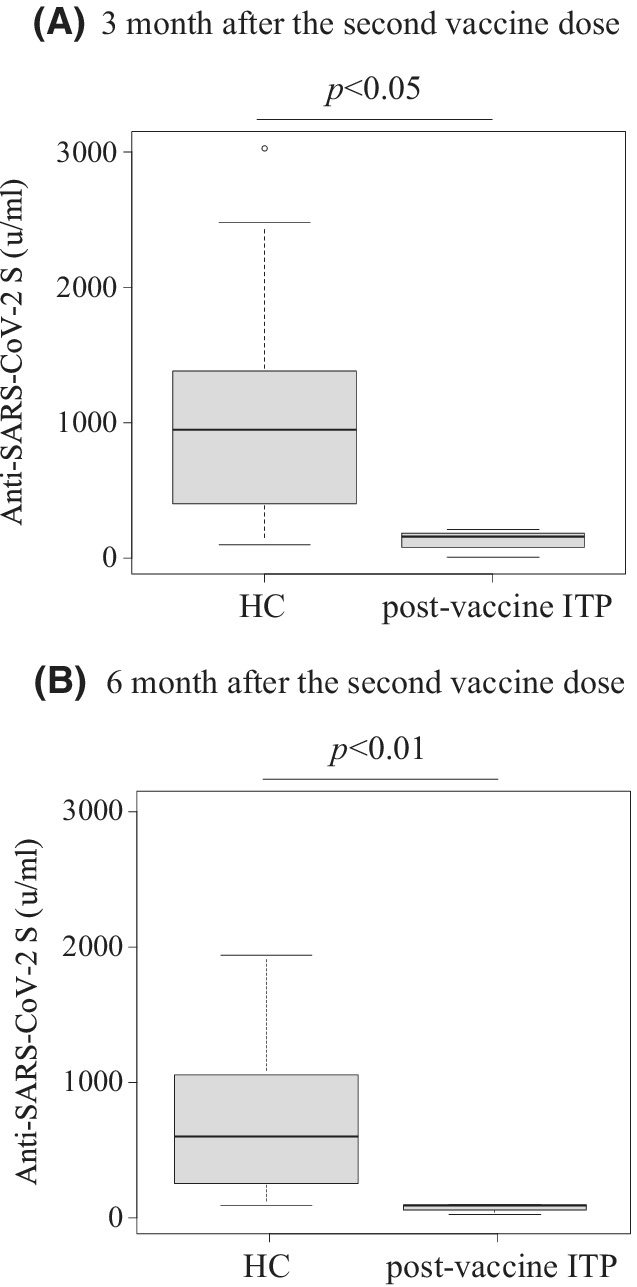
Anti‐SARS‐CoV‐2S antibody titres after vaccination in patients with post‐vaccine ITP exacerbation treated with corticosteroids. (A) Anti‐SARS‐CoV‐2S antibody titres in HCs (*n* = 28) and in patients with post‐vaccine ITP exacerbation treated with corticosteroids (*n* = 3) at 3 months after the second vaccine dose. (B) Anti‐SARS‐CoV‐2S antibody titres in HCs (*n* = 24) and in in patients with post‐vaccine ITP exacerbation treated with corticosteroids (*n* = 3) at 6 months after the second vaccine dose. HC, healthy controls; ITP, immune thrombocytopenia; SARS‐CoV‐2S, severe acute respiratory syndrome coronavirus‐2 spike. The boxes show interquartile range, centre line shows the median.

A third dose of mRNA vaccine was reported to improve humoral immunity against SARS‐CoV‐2 and to be associated with seroconversion even in immunocompromised patients.^12^, ^14^ In our study, two patients developed ITP after the third dose (UPN1 and UPN3). These patients showed a marked increase of antibody titres after the third dose and rescue treatments were successful. On the other hand, another patient (UPN5) who developed de novo ITP after the second dose showed a marked increase of the antibody titre without developing ITP after the third dose. Based on very limited studies^4^, ^6^ and our results, it is recommended that patients with ITP should undergo at least the initial vaccination and that subsequent vaccination should be considered more carefully in patients who developed ITP after COVID‐19 vaccination.

There is still not enough evidence to inform the optimal management of a patient presenting with new or relapsed ITP during the COVID‐19 pandemic.^15^ Depending on the situation, the use of thrombopoietin receptor agonists and intravenous immunoglobulin may be considered.^15^ Other immunosuppressive treatments, including rituximab, may reduce vaccine response, as may corticosteroids.

In conclusion, careful monitoring of the platelet count after vaccination should be performed for patients with pre‐existing ITP. In patients with post‐vaccine ITP exacerbation, standard treatment with corticosteroids could abrogate the chance of acquiring anti‐SARS‐CoV‐2 antibody.

## AUTHOR CONTRIBUTIONS

Akio Mori designed the study, analysed the data and wrote the manuscript. Masahiro Onozawa and Takanori Teshima revised and approved the manuscript. Mirei Kobayashi, Shihori Tsukamoto, Takashi Ishio, Emi Yokoyama, Koh Izumiyama, Makoto Saito and Masanobu Morioka performed recruitment and treatment of patients and provided critique to the manuscript. Haruna Muraki performed experiments and provided critique to the manuscript. Takeshi Kondo designed and supervised the study and approved the manuscript. All authors read and approved the final manuscript.

## CONFLICTS OF INTEREST

The authors declare that they have no conflict of interest.

## CLINICAL TRIAL REGISTRATION

This study was part of a prospective observation study (UMIN000045267) and it was conducted in compliance with ethical principles based on the Helsinki Declaration and was approved by the Institutional Review Board of Aiiku Hospital.

## PATIENT CONSENT STATEMENT

Written informed consent was obtained from all individuals included in the study.

## Data Availability

The datasets generated and/or analysed during the present study are available from the corresponding author upon reasonable request.
